# Determining the Psychometric Properties of the Turkish Version of the Positive Caregiving Experience Scale in Dementia

**DOI:** 10.1002/gps.70063

**Published:** 2025-03-13

**Authors:** Mükerrem Kabataş Yıldız, Ayşe Çal

**Affiliations:** ^1^ Department of Health Care Services Ondokuz Mayıs University Vocational School of Health Services Elder Care Program Samsun Turkey; ^2^ Ankara Medipol University School of Health Sciences Ankara Turkey

**Keywords:** care experience, caregiver, dementia, validity and reliability

## Abstract

**Background:**

Caring for individuals with dementia presents significant challenges for caregivers. However, positive experiences such as personal growth and emotional satisfaction play a vital role in fostering resilience and improving caregiving outcomes. This study highlights the need for a culturally relevant tool to assess these positive feelings, focusing on the adaptation and validation of the Dementia Caregiver Positive Feeling Scale for Turkish caregivers.

**Objectives:**

This study aimed to carry out the Turkish validity and reliability study of the 21‐item Dementia Caregiver Positive Feeling Scale.

**Methods:**

This methodological study was conducted between February and December 2023 with 200 caregivers of dementia patients receiving treatment at the education and research hospital in Samsun, Türkiye. Data were collected using an individual information form and the 21‐item Dementia Caregiver Positive Feeling Scale. Data analysis was performed using SPSS 22.0 and Amos 22.0 software. Cronbach's alpha reliability coefficient was calculated, and exploratory factor analysis and confirmatory factor analysis were conducted to test the construct validity of the scale.

**Results:**

The average age of caregivers was 45.18 ± 12.02 (min = 18, max = 78), and 78.0% were female. The average caregiving duration was 4.07 ± 3.00 (1–18) years. The total Cronbach's alpha value of the scale is 0.80. Exploratory factor analysis revealed a KMO coefficient of 0.756 and a Bartlett's test result of *χ* ^2^ = 960.382, *p* < 0.001. Factor loadings ranged from 0.32 to 0.61 and the total variance explained was 47.232. Confirmatory factor analysis supported the scale's 20‐item, four‐factor structure, with fit indices indicating an acceptable model fit: RMSEA 0.07, GFI 0.85, AGFI 0.80, CFI 0.77 and *χ*2/sd 2.15 (*p* < 0.001).

**Conclusions:**

The Dementia Caregiver Positive Feeling Scale has been determined to be a valid and reliable instrument for the Turkish community. It serves as a valuable tool that can be used in research evaluating the positive experiences of caregivers of dementia patients.

**Summary**

- The Dementia Caregiver Positive Feeling Scale was successfully adapted and validated for Turkish caregivers, ensuring its cultural relevance.
- Exploratory and confirmatory factor analyses confirmed a 20‐item, four‐factor model, indicating a stable psychometric structure.
- This validated tool can help researchers and clinicians assess positive caregiving experiences among dementia caregivers in Türkiye.
- The scale provides a valuable contribution to dementia caregiving research, emphasizing the importance of positive emotional experiences in caregiving.

## 1 Introduction

The rapid increase in the aging population has led to a corresponding rise in the prevalence of dementia, a condition that has adverse effects on cognitive functions and daily activities [1]. According to the World Health Organization [2], over 55 million people worldwide have been diagnosed with dementia. Dementia has become a significant public health concern as one of the leading causes of disability and dependency among the elderly and ranking seventh among causes of death globally. The WHO identifies dementia as a public health priority [2]. In Türkiye, while the exact prevalence of dementia remains unknown, studies estimate it to range between 16.8% and 20.0% [3, 4]. Dementia is an umbrella term used for a variety of conditions that affect memory, thinking, and the ability to perform daily tasks [2]. Individuals diagnosed with dementia often receive outpatient treatment, with their care and disease management primarily undertaken at home by family members [5]. Therefore, family members play a significant role in the care of individuals with dementia [6].

Family caregivers of individuals with dementia take on a multifaceted role that extends beyond basic caregiving tasks. They are actively involved in managing medical treatments, providing emotional support, ensuring safety, maintaining communication, managing symptoms, and facilitating health checks [7]. However, caregiving is often associated with significant challenges, including physical and mental health issues, social and economic burdens, and deterioration in family relationships for caregivers [8]. The care of individuals with dementia is very demanding and can lead to a wide range of emotional experiences for caregivers [9]. While much of the literature often focuses on negative emotions and responsibilities in the caregiving experience, there is a growing recognition of the importance of positive emotions in the caregiving experience [9, 10, 11]. Positive perceptions of the caregiving process by family members who take care of the individual with dementia at home has been identified as critical factors in sustaining caregiving efforts [11, 12].

Recognizing the positive aspects of caring for a person with dementia can enhance caregivers' health, well‐being, family relationships, and the quality of care they provide [6, 9]. Family caregivers can experience various rewarding emotions, such as feeling needed and useful, which contribute to their satisfaction and sense of purpose [13, 14]. Positive emotions such as happiness, satisfaction, and the ability to set meaningful goals play an important role in mitigating the negative outcomes of caregiving. As a result, both the caregiver's and the person with dementia's quality of life and overall well‐being are improved [15, 16]. Considering the emotional interactions inherent in dementia care, recognizing and evaluating the positive emotions caregivers experience becomes essential [17]. To this end, an appropriate scale is needed to measure these positive aspects of caregiving. While the Turkish version of the Scale for Positive Aspects of Caregiving Experience developed by [18] provides a general measure of caregiving experiences, it does not address the unique needs of dementia family caregivers. The scale is suitable for general caregiving contexts but lacks the focus required to evaluate the challenges and positive emotional experiences of dementia caregivers, such as managing cognitive decline and behavioral changes. The Dementia Caregiver Positive Feeling Scale, originally developed by Fuju and colleagues in 2021, offers a significant advantage in this context. Specifically designed to assess and promote positive emotions in dementia caregivers, the scale captures the unique emotional and caregiving dynamics of dementia care. This focused, reliable, and valid tool is vital for understanding and supporting the positive experiences of dementia caregivers. Based on this need, the present study aims to adapt the Dementia Caregiver Positive Feeling Scale into Turkish and evaluate its psychometric properties, including validity and reliability. This may provide a culturally appropriate tool for assessing positive caregiving experiences among Turkish family caregivers of individuals with dementia. To achieve this goal, the study seeks to answer the following research questions:

1. Is the Turkish version of the Dementia Caregiver Positive Feeling Scale a valid measurement tool?
2. Is the Turkish version of the Dementia Caregiver Positive Feeling Scale a reliable measurement tool?

## 2 Method

### 2.1 Study Type

This study was conducted using a methodological research design.

### 2.2 Study Setting and Time

This study was conducted at a tertiary education and research hospital located in Samsun, Türkiye, between February and December 2023.

### 2.3 Population and Sample

The study population consisted of caregivers of dementia patients receiving treatment at an education and research hospital in Samsun province. According to Karagöz [19], a minimum of five participants per item is recommended to conduct factor analysis in validity and reliability studies. To ensure a clearer assessment of the invariance of the 21‐item scale, the study planned to include 9–10 caregiver family members per item. Consequently, a total of 200 caregiver family members were included in the sample.

### 2.4 Inclusion Criteria

The study included caregivers of individuals with dementia receiving treatment at a tertiary care hospital in Samsun. The inclusion criteria for the participants were being over 18 years of age, literate, capable of understanding and answering the questions posed, and willing to participate in the study.

### 2.5 Data Collection Instruments

The study utilized the Individual Information Form, including questions about the socio‐demographic characteristics of the family members caring for individuals with dementia, and the 21‐item version of the Dementia Caregiver Positive Feeling Scale to collect data.

**Individual Information Form:** The 24‐item form, developed by the researchers based on the literature [14, 15], captures the socio‐demographic characteristics of the family members caring for individuals with dementia and the characteristics related to the dementia disease and the caregiving process.

**Dementia Caregiver Positive Feeling Scale ‐21 Item Version:** This scale was developed to assess the positive aspects of caregiving for individuals with dementia. It was also designed to help family caregivers recognize and appreciate the positive experiences that arise during the caregiving process. The scale consists of 21 items under four subscales, measured using a four‐point Likert scale. Responses are scored as follows: 1 = Strongly Disagree, 2 = Disagree, 3 = Agree, and 4 = Strongly Agree. Higher scores indicate greater positive caregiving experiences among family members. The Cronbach's Alpha coefficient of the scale is 0.92 [20].

### 2.6 Adapting the Scale to Turkish

In scale adaptation, it is necessary to use the sentence structures and expressions that are most suitable for the target language and to ensure that the content aligns with the cultural context [19]. For this purpose, the scale was translated into Turkish separately by five Japanese linguists. After the scale was translated into Turkish, the researchers collaborated to develop the Turkish version of the scale. A different linguist proficient in both Turkish and Japanese performed a back‐translation of the Turkish version into Japanese to verify the accuracy and consistency of the translation.

### 2.7 Content Validity of the Scale

For scales involving translation, it is recommended to obtain the opinion of at least three experts to ensure equivalence with the original scale [19, 21]. In the study, opinions were collected from eight experts, including five public health nursing faculty members, two psychiatric nursing faculty members, and one elderly care program faculty member. The experts were provided with the original version of the scale, its English translation (sent by the original developer), and the Turkish translation. Based on their feedback, the scale items were revised to ensure accuracy and cultural relevance. The language content validity of the scale was assessed using the Davis technique [22]. Experts evaluated each item using the following ratings: *a* = Appropriate, *b* = Item needs minor revision, *c* = Item needs significant revisions, and *d* = Item is not appropriate. The content validity index (CVI) for each item was calculated by dividing the number of experts selecting options (a) and (b) by the total number of experts. A CVI value of 0.80 or higher was considered acceptable [22].

### 2.8 Reliability Analyses of the Scale

The reliability of the scale was assessed through analyses of internal consistency and stability. Cronbach's alpha, item‐total score correlations, and item‐subscale total score correlations were used for internal consistency. A Cronbach's alpha coefficient between 0.60 and 0.79 indicates moderate reliability, while a value between 0.80 and 1.00 reflects high reliability [23]. It is also recommended that the correlation coefficients for item‐total and item‐subscale total scores should be at least 0.20 to be considered acceptable [24, 25]. To further support the internal consistency analysis, Hotelling's *T*‐Squared test and Intraclass Correlation Coefficient (ICC) analysis were also conducted.

### 2.9 Validity of the Scale Structure

The validity of the scale structure was examined using exploratory and confirmatory factor analysis. The adequacy and suitability of the dataset for factor analysis were assessed using the Kaiser‐Meyer‐Olkin (KMO) coefficient and the Bartlett's Test of Sphericity. A *p*‐value of less than 0.05 for Bartlett's Test and a KMO value greater than 0.60 are recommended to confirm the suitability of the data for factor analysis. The principal components analysis and the varimax rotation method were applied to determine the validity of the scale structure. Eigenvalues of 1 or higher were used to identify the most appropriate factor structure and the number of factors [19, 26, 27, 28]. Experts highlight that a minimum factor loading of 0.30 is required to determine which items align with which factors [19, 29]. In this study, the threshold for factor loadings was set at 0.30 to establish item‐factor assignments [19, 26, 27, 28].

Another method used to evaluate structural validity is confirmatory factor analysis (CFA). As part of the CFA, several fit indices were examined, including Pearson Chi‐Square, degrees of freedom (DF), root‐mean‐square error of approximation (RMSEA), goodness of fit index (GFI), comparative fit index (CFI) and adjusted goodness of fit index (AGFI). For an acceptable model fit, it is suggested that the value obtained by dividing the chi‐square value by the degree of freedom should be less than five, RMSEA should be below 0.080, GFI and AGFI values should be 0.80 or higher, and other fit indices should also be above 0.90 [19, 30, 31, 32, 33].

### 2.10 Data Analysis

The descriptive characteristics of caregivers (number and frequency), Cronbach's alpha reliability analysis, exploratory factor analysis, Hotelling's T‐Squared analysis, and Intraclass Correlation tests were analyzed using SPSS for Windows Version 22.0 (IBM Corporation, Armonk, NY, USA). Confirmatory factor analyses and modifications of the scale were performed using AMOS 22.0 (Amos Development Corporation, Meadville, PA, USA).

### 2.11 Ethical Considerations

Before initiating the research, permission was obtained from the author of the original scale for its adaptation and use in Turkish. Ethical approval was granted by the ethics committee (Date: 30.12.2022, Decision number: 2022–1167), and institutional permission was obtained from the hospital where the study was conducted. During data collection, participants were informed about the purpose of the study, the research team was introduced, and it was assured that participation was entirely voluntary. The participants were also informed that they could withdraw from the study at any time, their information would be kept confidential, and the research results would be used only for scientific purposes. Informed consent was obtained from all participants. The study adhered to the ethical principles in the Helsinki Declaration at all stages of the study.

## 3 Results

### 3.1 Demographic Characteristics of the Sample

The average age of the caregivers who participated in the study was 45.18 ± 12.02 (min = 18, max = 78). Among the participants, 78.0% were female and married, 60.5% had a nuclear family structure, 43.0% had a primary school education or lower, 30.5% were employed, 66.0% had an income equal to their expenses, and 43.0% lived in a town. The average duration of care was 4.07 ± 3.00 years (min = 1, max = 18), and the daily care time was 10.33 ± 7.30 h (min = 1, max = 24). The average age of the individuals they cared for was 80.99 ± 7.94 (min = 63, max = 105), 54.5% were female, and 67.0% were single (Table 1).

**TABLE 1:** Demographic characteristics of the students.

Characteristics	$\bar{x}$ ± SD	Min‐max
Age	45.18 ± 12.02	18–78
Age (patient)	80.99 ± 7.94	63–105
Year of caregiving	4.07 ± 3.00	1–18
Daily caregiving hours	10.33 ± 7.30	1–24

	*n*	%
Gender		
Female Male	156 44	78.0 22.0
Gender (patient)		
Female Male	109 91	54.5 45.5
Marital status		
Married Single	156 44	78.0 22.0
Marital status (patient)		
Married Single	66 134	33.0 67.0
Family type		
Nuclear Extended	121 79	60.5 39.5
Level of education		
Primary school and below Middle school High school University and above	86 37 46 31	43.0 27.5 23.0 15.5
Employment status		
Working Not working	61 139	30.5 69.5
Income status		
Income less than expenses Income equal to expenses Income higher than expenses	41 132 27	20.5 66.0 13.5
Place of residence		
City Town Village	66 86 48	33.0 43.0 24.0

### 3.2 Content Validity

The linguistic and content validity of the scale was assessed using the Davis technique [22]. Expert opinions were evaluated on a four‐point scale: *a* = Appropriate, *b* = Item should be slightly reviewed, *c* = Item should be seriously reviewed, and *d* = Item is not appropriate. The content validity index for each item was calculated by dividing the number of experts selecting (a) and (b) options by the total number of experts. A CVI of 0.80 or higher was considered acceptable [22]. For linguistic scope validity, the index values obtained from seven experts were 1.00 for all items. Therefore, it can be concluded that the scale ensured content validity.

### 3.3 Descriptive Statistics for the Dementia Caregiver Positive Feeling Scale

The average total score on the Dementia Caregiver Positive Feeling Scale was 3.2 ± 0.3 (min = 1.7, max = 4.0). Subscale scores were as follows: the meaning of caregiving = 3.3 ± 0.4 (min = 1.2, max = 4.0); mastery in caregiving = 3.1 ± 0.4 (min = 1.7, max = 4.0); positive emotions in care experience = 3.4 ± 0.5 (min = 2.0, max = 4.0); and psychological resilience in caregiving = 3.2 ± 0.4 (min = 2.0, max = 4.0) (Table 2).

**TABLE 2:** Score distribution of the Dementia Caregiver Positive Feeling Scale.

Scale and sub‐scales	$\bar{x}$ ± SD	Min	Max	Cronbach *α*
Scale total	3.2 ± 0.3	1.7	4.0	0.80
Meaning of care giving	3.3 ± 04	1.2	4.0	0.73
Mastery in care giving	3.1 ± 0.4	1.7	4.0	0.65
Positive emotions in care experience	3.4 ± 0.5	2.0	4.0	0.60
Psychological resilience in caregiving	3.2 ± 0.4	2.0	4.0	0.62

### 3.4 Item Analysis

The items were analyzed for their relationship with the total scale score following the scope validity assessment. One item (item 8 in the original scale) with a correlation value below 0.20 was excluded from the scale. The corrected item‐total correlation values for the remaining 20 items were determined to be in the range of 0.21–0.61 (Table 3).

**TABLE 3:** Validity and reliability results for the dementia caregiver positive feeling scale.

Factors and items		Factor loadings	$\bar{x}$ + SD	Item‐ total score correlation	*t* (Lower % 27‐ upper %27)	*p* value (Lower % 27‐ upper %27)	Cronbach alfa if item deleted	Variance explained (%)
Factor 1: Meaning of care giving (*α* = 0.73)								14.379
1	Taking care of the person I'm responsible for makes me happy.	0.579	3.1 ± 0.6	0.376	−5.737	< 0.001	0.765	
2	The bond between the person I care for and me has strengthened.	0.503	3.2 ± 0.7	0.390	−5.136	< 0.001	0.764	
4	The existence of the person I care for makes me happy.	0.613	3.3 ± 0.6	0.536	−7.778	< 0.001	0.756	
14	When I see the person I care for smile, I become happy.	0.518	3.6 ± 0.5	0.574	−11.702	< 0.001	0.756	
21	When needed, I can reach experienced health personnel and social support systems on the subject of dementia.	0.364	3.1 ± 0.7	0.327	−5.565	< 0.001	0.768	
Factor 2: Mastery in care giving (*α* = 0.65)								11.446
5	I Learned things from the person I care for.	0.351	3.2 ± 0.7	0.354	−5.668	< 0.001	0.766	
6	I Started to think that my life is meaningful.	0.316	3.2 ± 0.8	0.321	−5.684	< 0.001	0.769	
9	I am grateful to the person I care for.	0.587	3.0 ± 0.7	0.332	−6.039	< 0.001	0.768	
12	People around me have become more conscious about dementia.	0.546	3.1 ± 0.7	0.306	−5.471	< 0.001	0.770	
17	I try to improve myself to be able to give better care.	0.383	3.2 ± 0.6	0.446	−5.852	< 0.001	0.762	
19	Getting help in care allowed me to take some time for myself. *Those who do not get help in care, please definitely choose I do not agree (1).	0.318	2.6 ± 1.2	0.283	−7.056	< 0.001	0.780	
20	My family has become more conscious about dementia.	0.497	3.4 ± 0.6	0.415	−7.289	< 0.001	0.763	
Factor 3: Positive emotions in care experience (*α* = 0.60)								11.387
3	Providing care is a return of the spiritual debt to the person receiving care.	0.481	3.0 ± 0.6	0.214	−4.762	< 0.001	0.783	
15	I am happy when I see the person I care for can smoothly carry out daily activities (dressing, eating, etc.) on their own.	0.464	3.6 ± 0.6	0.313	−5.339	< 0.001	0.769	
16	I am happy when I see the person I am caring for smile.	0.548	3.5 ± 0.6	0.377	−7.503	< 0.001	0.766	
18	I feel better when I talk to someone in the same situation as me.	0.470	3.4 ± 0.7	0.249	−5.899	< 0.001	0.773	
Factor 4: Psychological resilience in caregiving (*α* = 0.62)								10.021
7	I Learned to be patient through caregiving.	0.499	3.6 ± 0.5	0.378	−7.226	< 0.001	0.766	
10	When the person I care for asks the Same question over and over, I respond as if I'm hearing it for the first time.	0.574	3.0 ± 0.7	0.271	−5.812	< 0.001	0.772	
11	I have become patient in listening to what the person I care for is telling.	0.571	3.2 ± 0.6	0.452	−8.381	< 0.001	0.761	
13	I can cope with the different behaviors of the person I care for (aimless wandering, forgetfulness, etc.).	0.396	3.2 ± 0.6	0.242	−3.985	< 0.001	0.773	
Total Cronbach's alpha (*α* = 0.80)								
Total variance explained (%)								47.232

### 3.5 Exploratory Factor Analysis

The exploratory factor analysis revealed that the Dementia Caregiver Positive Feeling Scale had a KMO coefficient of 0.756, and the result of the Bartlett's test was *χ* ^2^ = 960.382, *p* < 0.001. The factor loadings of the scale ranged from 0.32 to 0.61, and the total explained variance was 47.232% (Table 3).

**FIGURE 1:**
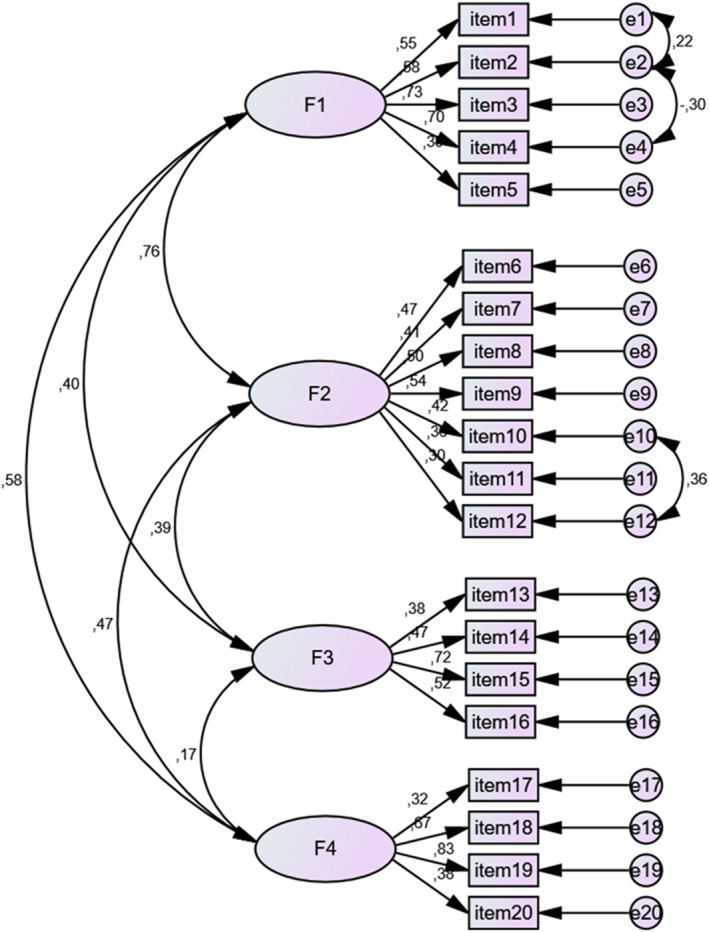
Diagram of Confirmatory Factor Analysis for Dementia Caregiver Positive Feeling Scale Confirmatory factor analysis.

Confirmatory factor analysis determined that the scale's 20‐item, four‐subscale structure was significant (*p* < 0.001) based on structural equation modeling results. Modifications were applied to improve model fit, including identifying variables that reduced the fit and forming new covariances among residuals with high covariance (e.g., e1–e2, e2–e4, e10–e12). After these adjustments, the revised fit indices met the acceptable threshold values, as shown in Table 4. The goodness‐of‐fit indices for the first‐order multifactorial analysis of the scale were as follows: RMSEA = 0.07, CFI = 0.77, GFI = 0.85, AGFI = 0.80, and *χ* ^2^/df = 2.15 (*p* < 0.001) (Table 4). The results of the first‐order multifactor CFA of the Dementia Caregiver Positive Feeling Scale are presented in Figure 1.

**TABLE 4:** Pre‐modification and post‐modification multifactorial confirmatory factor analysis results of the dementia caregiver positive feeling scale before and after modification.

Goodness of fit measurements	Perfect fit criteria	Acceptable fit criteria	Pre‐modification	Post‐modification
CMIN/Df	0 ≤ *χ* ^2^/df ≤ 3	3 ≤ *χ* ^2^/df ≤ 5	2.39	2.15
GFI	0.90 ≤ GFI	0.80 ≤ GFI	0.83	0.85
AGFI	0.90 ≤ AGFI	0.80 ≤ AGFI	0.78	0.80
CFI	0.95 ≤ CFI	0.85 ≤ CFI	0.72	0.77
RMSEA	0.0 ≤ RMSEA ≤ 0.05	0.06 ≤ RMSEA ≤ 1.0	0.08	0.07

Accordingly, the scale's structure, consisting of four subscales and 20 items, was accepted. The factorial loadings of the scale items were in the range of 0.32–0.61.

### 3.6 Reliability Analysis

The total Cronbach's alpha internal consistency reliability coefficient value of the Dementia Caregiver Positive Feeling Scale was 0.80. The item‐total score correlation coefficients of the scale ranged from 0.17 (lowest) to 0.57 (highest) (Table 3).

The Hotelling's *T*‐Squared test result was significant (*F* = 19.214; *p* < 0.05). According to this result, the scale measured the intended construct at a statistically significant level (Table 5).

**TABLE 5:** Data related to Hotelling's *T*‐Squared analysis of the Dementia Caregiver Positive Feeling Scale.

Hotelling's T‐Squared	F	df1	df2	*p*
401.362	19.214	19	181	< 0.001

According to the Intraclass Correlation Coefficient test, regarding the order and structural characteristics of the scale items, it was found that both individual items (*r* = 0.148) and average measurements (0.777) were valid and reliable (*p* < 0.05) (Table 6).

**TABLE 6:** Intraclass correlation coefficient test results for the self‐advocacy in dementia caregiver positive feeling scale.

	Intraclass correlation	95% confidence interval		F Statistics			
		Lower limit	Upper limit	Test value	df1	df2	*p*
Single measures	0.148	0.19	0.185	4.476	199	3781	< 0.001
Average measures	0.777	0.729	0.819	4.476	199	3781	< 0.001

### 3.7 Structure and Evaluation of the Scale

The Dementia Caregiver Positive Feeling Scale is a measurement tool designed to highlight the positive aspects of caring for individuals with dementia. The scale consisted of 20 items under four subscales, with no negative statements included. The subscales of the scale are as follows “Meaning of caregiving” (items 1, 2, 4, 14, and 21), “Mastery in caregiving” (items 5, 6, 9, 12, 17, 19, and 20), “Positive emotions in caregiving experience” (items 3, 15, 16, and 18), and “Psychological resilience in caregiving” (items 7, 10, 11, and 13). The scale uses a 4‐point Likert format scored as follows: 1 = Strongly disagree, 2 = Disagree, 3 = Agree, and 4 = Strongly agree. The total and subscale score averages range from a minimum of 1 to a maximum of 4. Higher scores from the scale indicate that the positive aspects of caring for dementia patients were high. For the evaluation of the scale, the arithmetic mean of the responses given to the items within the total scale and its subscales is calculated. In this study, which focused on the Turkish adaptation of the scale, the Cronbach's alpha coefficient of the total scale was 0.80, while the coefficients for the subscales ranged between 0.60 and 0.73.

## 4 Discussion

The Dementia Caregiver Positive Feeling Scale, which we have validated and verified, evaluates the positive aspects of caregiving for individuals with dementia. The scale emphasizes the universal importance of positive emotions in the caregiving process [20]. Recognizing these positive emotions is crucial for maintaining caregiving efforts and alleviating caregiver burden [17]. Therefore, this measurement tool, which measures the positive emotional experiences of caregivers, is both essential and highly relevant in caregiving research and practice.

One of the important steps in methodological research where items are evaluated is item‐total correlations. In this study, the item‐total correlations of the scale were found to be above the threshold value of 0.20. This appropriate range of item correlations indicates that participants correctly understand the statements on the scale and respond objectively, and that the scale exhibits high item discriminability [24, 25].

The scale has a four‐dimensional structure consisting of 20 items. For exploratory factor analysis (EFA), which is used to determine the scale's structure, an adequate sample size is required. In this study, sample adequacy was evaluated using the KMO coefficient, which was found to be 0.756. KMO values between 0.80 and 0.90 are considered “very good” [34, 35]. Therefore, an adequate sample size for factor analysis has been reached in this study. In addition, the Bartlett's test of sphericity was statistically significant, confirming that there is a relationship between the scale items [36]. The factor loadings of the scale items exceeded the threshold value (> 0.30), and item adequacy was provided [37, 38, 39]. In the original article by Fuju [20], the factor loadings are between 0.30 and 0.74. In addition, the scale achieved a variance explanation ratio of %47.2, which reflects the robustness of its structure and the quality of the items [38, 39].

Multiple fit indices were used in the CFA to evaluate the construct validity of the scale. The accuracy of the scale's structure was assessed by considering all indices together [40, 41, 42]. In the literature, the fit is considered acceptable when *χ*2/df is between 3 and 5, RMSEA is between 0.05 and 1, and GFI and AGFI values are 0.80 and above [19, 31, 32, 33, 42, 43, 44]. Fuju [20] confirmed the scale's four‐factor and 21‐item structure in their study. In the current study, the Dementia Caregiver Positive Feeling Scale demonstrated a good fit for the four‐factor, 20‐item structure, with high fit index values. These results confirm that the scale is suitable for assessing the positive aspects of caregiving for individuals with dementia.

In this study, the Cronbach's alpha value for the total scale was found to be 0.80. The Cronbach's alpha values for the subscales are between 0.60 and 0.73. In the original study, the Cronbach's alpha value for the total scale was reported as 0.92, and for the subscales, it was between 0.74 and 0.88 [20]. In the literature, if the Cronbach's alpha coefficients are between 0.60 and 0.79, the measurement tool is considered relatively reliable, and if they are between 0.80 and 1, the tool is considered highly reliable [23].

The scale, consisting of 20 items, is concise and user‐friendly, making it easy for caregivers to complete. Another positive aspect of the scale is that it gives messages about the positive aspects of caring for individuals with dementia. When individuals who care for dementia patients read the scale items, it is anticipated that they will gain curiosity and awareness about the positive effects of caregiving. In addition, individuals who respond to the scale may start to notice the positive effects of caregiving on themselves.

### 4.1 Limitations

A limitation of the study is that due to its design, test‐retest reliability could not be confirmed.

## 5 Conclusion

The study concludes that the Dementia Caregiver Positive Feeling Scale is a valid and reliable tool for use in Turkish society. The scale has a validated structure, consisting of 20 items, organized into a total score and four subscales. It could be recommended to increase its widespread effect by re‐validating and verifying its reliability on larger samples and among diverse cultural groups.

## Ethics Statement

To conduct the research, approval was obtained from the Ondokuz Mayıs University Social and Human Sciences Ethics Committee (Date: 30.12.2022, Decision number: 2022–1167). Permission was also obtained from the developers of the scales used in the study. Informed consent was acquired from participants by adding an explanation to the data collection form, clarifying the research team and purpose, emphasizing the voluntary nature of participation, stating that participants could withdraw from the study at any time, ensuring the confidentiality of their information, and specifying that the data would be used solely for scientific purposes. Throughout all stages of the study, ethical principles outlined in the Helsinki Declaration were adhered to.

## Conflicts of Interest

The authors declare no conflicts of interest.

## Clinical Contribution

The adapted Turkish version of the scale is a valuable measurement tool in clinical research, specifically designed to evaluate the positive aspects of caregiving for individuals with dementia. Nurses and academics working in the fields of aging, dementia and home care can plan initiatives to empower both patients and caregivers by revealing the positive aspects of caregiving in their research.

## Data Availability

The data that support the findings of this study are available from the corresponding author upon reasonable request.
